# YOLO-RGDD: A Novel Method for the Online Detection of Tomato Surface Defects

**DOI:** 10.3390/foods14142513

**Published:** 2025-07-17

**Authors:** Ziheng Liang, Tingting Zhu, Guang Teng, Yajun Zhang, Zhe Gu

**Affiliations:** 1 College of Mechanical and Electronic Engineering, Nanjing Forestry University, Nanjing 210037, China; 19515622659@163.com (Z.L.); tengg@njfu.edu.cn (G.T.); zhangyj@njfu.edu.cn (Y.Z.); 2 College of Agricultural Science and Engineering, Hohai University, No. 1 Xikang Road, Nanjing 210098, China; zhegu2018@hhu.edu.cn

**Keywords:** defect detection, tomato, YOLOv12, online defection, dynamic convolution

## Abstract

With the advancement of automation in modern agriculture, the demand for intelligence in the post-picking sorting of fruits and vegetables is increasing. As a significant global agricultural product, the defect detection and sorting of tomato is essential to ensure quality and improve economic value. However, the traditional detection method (manual screening) is inefficient and involves high labor intensity. Therefore, a defect detection model named YOLO-RGDD is proposed based on YOLOv12s to identify five types of tomato surface defects (scars, gaps, white spots, spoilage, and dents). Firstly, the original C3k2 module and A2C2f module of YOLOv12 were replaced with RFEM in the backbone network to enhance feature extraction for small targets without increasing computational complexity. Secondly, the Dysample–Slim-Neck of the YOLO-RGDD was developed to reduce the computational complexity and enhance the detection of minor defects. Finally, dynamic convolution was used to replace the conventional convolution in the detection head in order to reduce the model parameter count. The experimental results show that the average precision, recall, and F1-score of the proposed YOLO-RGDD model for tomato defect detection reach 88.5%, 85.7%, and 87.0%, respectively, surpassing advanced object recognition detection algorithms. Additionally, the computational complexity of the YOLO-RGDD is 16.1 GFLOPs, which is 24.8% lower than that of the original YOLOv12s model (21.4 GFLOPs), facilitating the model’s deployment in automated agricultural production.

## 1. Introduction

Tomatoes, being an essential global cash crop, boast massive annual production. However, due to their exposure to bacteria and viruses [1], the surface of tomatoes will have different categories and degrees of defects (e.g., hyperplasia, cracks, white spots, etc.), significantly reducing their value as a commodity and the security of their storage. Traditional manual sorting methods [2] rely on experience and are inefficient, making it challenging to meet the demands of large-scale production. In contrast, existing machine vision-based detection methods are still deficient in multi-class complex defect-type recognition [3], small-target-feature capture, etc. [4], which restricts the practical application of agricultural automation technology.

Deep learning-based target detection is mainly divided into two categories: two-stage algorithms and single-stage algorithms [5]. Two-stage algorithms include ResNet [6], Fast-RCNN [7], etc., which show higher precision but poor real-time performance due to computational complexity limitations. Single-stage algorithms include SSD [8], EfficientDet [9], and the YOLO [10,11,12,13] series, where the YOLO series has become the mainstream choice in the industrial detection field due to its combination of efficiency and accuracy. In the example of object detection in fruits and vegetables, Gai et al. [14] added DenseNet and other modules into the backbone network of the YOLOv4 model to improve the average precision of cherry fruits by 0.15 times. Wang [15] and Chen [16] et al. solved the problem of fruit occlusion through the improvement of the YOLOv8n- and YOLOv5-based algorithms.

However, the existing YOLO model for fruit object detection mostly stays in the picking stage. In tomato object detection, Appe et al. [17] added the CBAM attention mechanism in the backbone network based on YOLOv5 in the tomato ripeness classification task and utilized the DIoU algorithm to achieve an average precision of 88.1%, which is an improvement of 2.2% compared to the original model. Zeng et al. [18] deployed a lightweight network on the mobile side to detect tomato ripeness. They reconfigured the backbone network of YOLOv5 using the bottleneck module of MobileNetV3, and the precision was only reduced by 2.16% while compressing the number of parameters of the model by 49.9%.

In addition to detection and deployment based on the ripeness of fruit or vegetables, some research exists on defect detection. For dual-channel fruit sorting detection involving apples, Fan et al. [19] using channel pruning and layer pruning methods, and the mean average precision increased to 93.7%, an increase of 2.2% compared to not using the methods. For a constructed pear dataset, Chen et al. [20] utilized the YOLOv4-P7 algorithm to achieve a mean average precision of 73.2%. Sato et al. [21] utilized YOLO for detecting cracks in cherry tomatoes, and crack defect detection for cherry tomatoes belongs to binary classification.

Although relevant research has been conducted on defect detection in fruits and vegetables as introduced above, there are still problems, such as low accuracy in detecting small defects and large computational complexity in detection models when detecting various defects of tomatoes. Therefore, a YOLO-RGDD model is proposed to detect five surface defects of tomatoes based on the YOLOv12 architecture. The contributions of this paper are as follows:

(1) To improve the detection accuracy of small defects, the RFEM and Slim-Neck structures have been introduced in YOLOv12 to enhance the feature extraction capability of small defects. The RFEM replaces the C3k2 module and A2C2f module in the backbone part, and the Slim-Neck consists of GSConv and VoVGSCSP modules in the neck part.

(2) To reduce the complexity and computational complexity, three improvements have been designed by sharing parameters for different branches in the backbone part, dynamic upsampling in the neck part, and dynamic convolution to select the number of parameters in the detection header, which is conducive to improving the detection efficiency of the model.

(3) A novel discrimination criterion, IoM, has been proposed to replace IoU to determine whether the prediction box correctly predicts various defects on tomatoes, which improves the accuracy of identifying irregular and discontinuous line shape defects, such as scars or gaps.

## 2. Data Collection and Preprocessing

### 2.1. Data Collection

**Figure 1:**
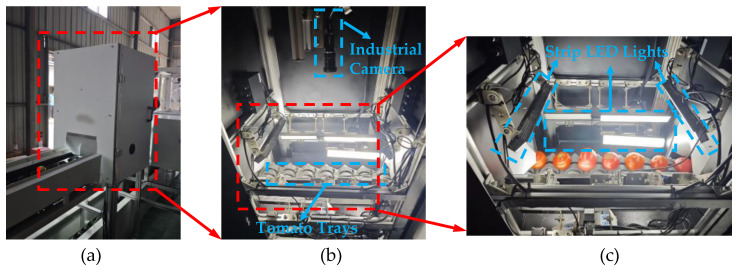
Data acquisition device for collecting tomato images. (**a**) The exterior of the complete device. (**b**) The internal structure of the acquisition device. (**c**) Scenario of collecting tomato images.

As shown in Figure 1, the optical acquisition system for collecting tomato images was set up in a closed dark box with a Hikvision MV-CS050–10GC color camera (5-megapixel net-mouth face array, Hangzhou, China) and a Hikvision MVL-KF1228M-12MPE lens (12 mm). The industrial camera was installed inside the box at the top, and two rows of LED lights were installed around the bottom to ensure that the light source was constant and stable. Tomatoes were rolled into the optical acquisition black box through the fruit tray to obtain our experimental original data without external environmental light interference. The resolution of tomato images was 916 pixels × 641 pixels. Finally, 3420 valid tomato images were collected.

### 2.2. Data Preprocessing

**Figure 2:**
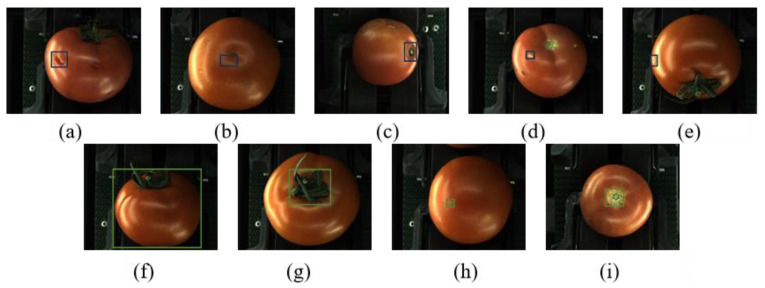
Sample of tomato labeling classification, surface defect labels: (**a**) gap: tomato with broken skin on surface; (**b**) scar: brownish, finely striped; (**c**) spoilage: severe defect, may have evolved from other defects, usually blackened; (**d**) white spot: white blotches produced by sunburn or insect damage; (**e**) dent: distinct from split; sunken surface without broken skin. Safety labels: (**f**) whole; (**g**) leaf; (**h**) calyx; (**i**) stem.

Five types of defects were proposed according to the style and degree of tomato surface defects: white spots, scars, dents, gaps, and spoilage. In addition, four types of labeling categories, whole, calyx, leaves, and stems, were designed to prevent the model from identifying the features carried by undamaged tomatoes as defects; therefore, they are also referred to as safety labels. An example of the nine labels is shown in Figure 2.

**Figure 3:**
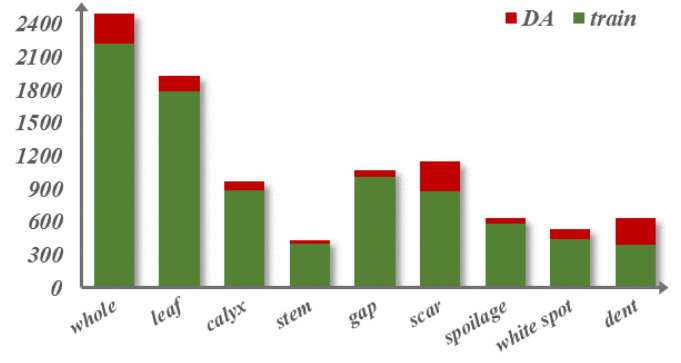
The distribution of every type in the training set. The green (train) represents the number of original images for each type, and the total is 2220; the red (data augmentation, DA) represents the number of augmented images of each type, and the total is 180.

In this experiment, LabelImg was used to label images, and the label file format was TXT. The labeled images were randomly divided proportionally to obtain 2220 training sets, 600 validation sets, and 600 test sets. To make the model learn the features of the defect categories more homogeneously, it should be ensured that the number of labels of each defect category is similar. After counting the number of labels in each of the existing training sets, 180 images were selected in the training set to balance the number of labels between different categories. For these 180 images, the data augmentation approach taken was horizontal and vertical inversion. The number of labels obtained statistically is shown in Figure 3. The enhanced training set reaches 2400 images, and the final ratio of the training sets, validation sets, and test sets is 4:1:1.

## 3. Method

YOLOv12 [22] is the latest version in the YOLO object detection algorithm series, designed by the Ultralytics team, which is a detector centered on the attention mechanism, mainly innovating the A2C2f module and following the C3k2 module proposed in YOLOv11 [23]. The core of the A2C2f module is the A2 attention mechanism, which divides the feature map into local regions to compute the attention, which reduces the computational complexity (from O(L^2^d) to O(L^2^d/2)) while maintaining a large sensory field, balancing speed and performance. The A2C2f module also applies the R-ELAN (Residual Efficient Layer Aggregation Network), which is an improved ELAN structure that introduces a new method of residual linkage and feature aggregation to solve the problem of instability in large model training while reducing the number of parameters and computation.

**Figure 4:**
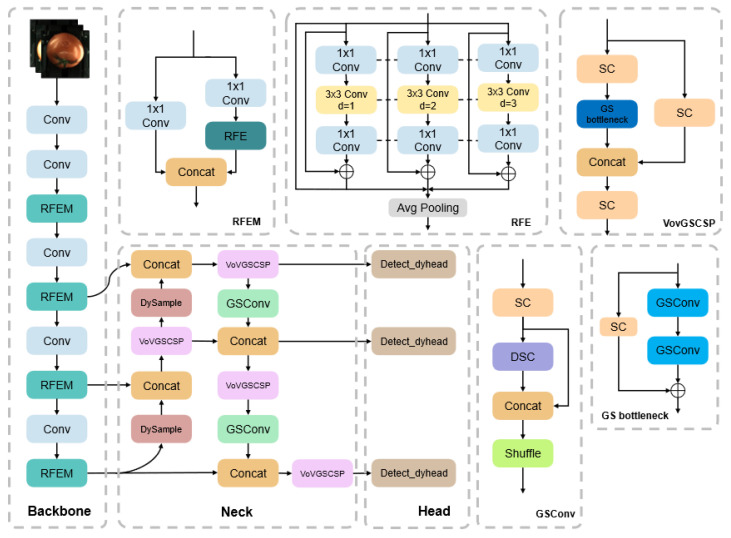
Network structure of YOLO-RGDD.

In terms of performance, YOLOv12 not only improves the detection precision but the computational complexity and parameters are also similar or less. In this study, a tomato surface defect detection model based on YOLO-RGDD was developed using YOLOv12 as the base network, and the model architecture is shown in Figure 4. The YOLO-RGDD model replaced the C3k2 module and A2C2f module in the original network backbone using the RFEM (Receptive Field Enhancement Module), which enlarges the effective sensory field without increasing the computational complexity by introducing dilated convolution and enhances the feature representation ability of the model for targets of different scales. The original up-sampling module was replaced by Dysample in the neck network. Dysample better handles the details and semantic information of the features by assuming that the input features are interpolated as a continuous feature map by bilinear interpolation and then resampling this continuous map by generating content-aware sampling points. At the same time, Slim-Neck was introduced. Its structure has a very important role in enhancing the feature expression, and with the dynamic convolution in the head network (Dy_detect), the rise of computational complexity can be avoided. The Dy_detect module is designed to keep the complexity low while increasing the parameters through a principle based on dynamic coefficient generation. The model significantly improves the detection precision while reducing the model complexity, which can be better applied to agricultural production scenarios.

### 3.1. RFEM Module

RFE, as a multi-branch expansion convolution design, is the main link of RFEM [24], enhancing the model’s ability to express features of targets at different scales and solving the problem where the detection target contains multiple scales despite the receptive field being insufficient, which causes the information of small targets to be lost. In the case of tomato surface defect detection, the environment is typically a conveyor belt, which is a single and constant environment. Tomato defects are complex and varied in size, and safety labels, such as calyx and pith, may be included as defective labels. The model must possess accurate feature expression capabilities for all types of different scale labels. Introducing the RFEM module enables the feature extraction of small targets to be effectively enhanced without increasing the model’s computational complexity.

**Figure 5:**
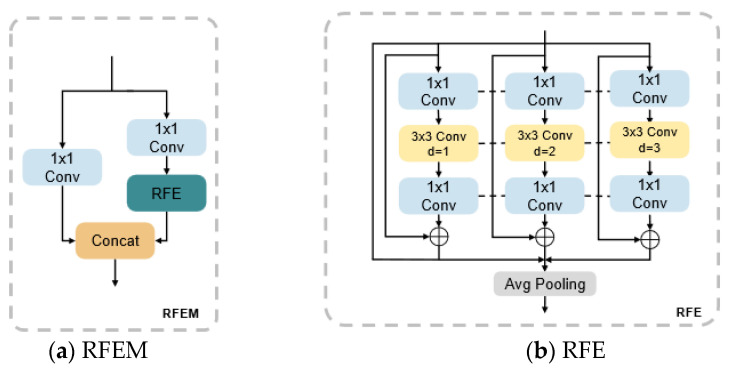
The structure of RFEM.

The network structure of RFEM is illustrated in Figure 5. Within RFE, it can be delineated into two components: expansion, convolution-based multi-branching and the aggregation weighting layer. Four parallel convolutional branches are employed in the expansion convolution-based multi-branching, where *d* signifies the expansion rate, specifically the spacing distance between elements in the convolution kernel. Additionally, residual connectivity is integrated between the dilation convolution branches to mitigate the issues of exploding and vanishing gradients during the training phase. The parameters of the convolution kernel can be shared among the various branches, significantly reducing the total number of parameters and thereby minimizing the potential risk of overfitting.

In the aggregation weighting layer, information is amassed from distinct branches and weighted accordingly to each branch of the features. The weights of each branch are dynamically adjusted through 1 × 1 convolution and average pooling, effectively balancing the contributions of different sensory wild features.

### 3.2. Dynamic Up-Sampling

Dynamic up-sampling (Dysample) [25] is different from the traditional up-sampling method, which uses a fixed-rule interpolation. Dysample dynamically decides which position of the input features to sample from by predicting the offset of each target point, which can better deal with the details of the features and the semantic information. In the case of surface defects in tomatoes, the three categories of white spots, scars, and spoilage are easy to confuse, and some of them need to be categorized based on the subtle gap to judge the categorization. Dysample grasps the detailed features of defects, which can improve the precision of detection without increasing the amount of computation.

**Figure 6:**
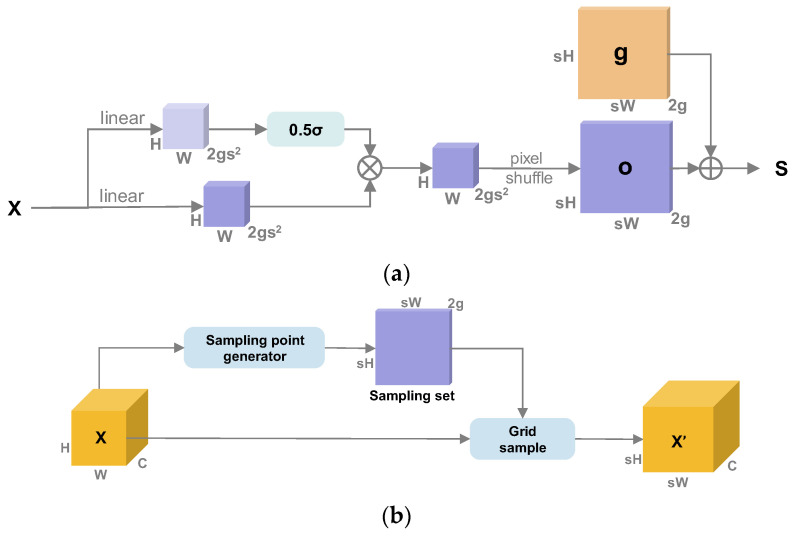
The structure of the Dysample module, where (**a**) is the upsampling module and (**b**) is the sample point generator with a dynamic scope factor.

The Dysample module is shown in Figure 6, and dynamic upsampling is divided into two main parts: generating the sample set S and the grid sample.

Generating the sample set S shows the workflow of the sample point generator, as in Figure 6a, where the input feature map X of size *C* × *H* × *W* is first generated by a linear layer, with an initial offset $o_{raw}$ calculated as:

(1) $$o_{raw}=Linear(X),o_{raw}\in R^{2gs^{2}\times H\times W}.$$

Then, the initial offset is deformed into a spatial resolution matching format o calculated as

(2) $$o=\mathrm{PixelShuffle}\left(o_{raw}\right),o\in R^{2g\times sH\times sW}.$$

A fixed bilinear interpolation network g is generated, and the offset o is superimposed on the standard network S as follows:

(3) $$S=g+o,o\in R^{2g\times sH\times sW},$$

Upsampling, as shown in Figure 6b, is the integration of the generating set *S* in the first part and the input feature map *X* by the grid sample to generate the final feature map X′ with size *C* × *sH* × *sW*, and the process is shown as follows:

(4) $$X^{\prime }={grid}_{sample}(X,S),X^{\prime }\in R^{C\times sH\times sW},$$

### 3.3. GSConv

GSConv [26] is the core of lightweight convolution, which is used as an alternative to standard convolution (SC) or deeply separable convolution (DSC) to improve feature representation while reducing computation. Meanwhile, GSConv is the base component of the VoV-GSCSP module, which optimizes feature extraction through the CSP structure. The combination of GSConv and VoV-GSCSP forms the Slim-Neck design, which is shown in Figure 4, and significantly reduces the number of parameters and the inference time while improving the precision at the detector neck.

**Figure 7:**
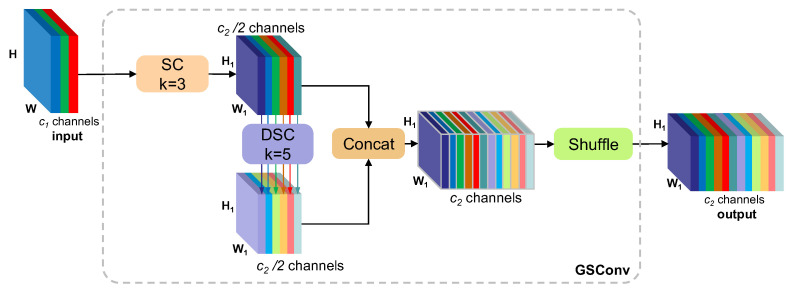
The structure of GSConv module.

The two components in GSConv, SC and DSC, are shown in Figure 7. SC is processed by a multi-channel convolution kernel to obtain the *C*_2_/2 × H × W feature map. DSC is by the channel-by-channel convolution for each input channel independently using a single-channel convolution kernel. A total of *C*_2_/2 convolution kernels are generated, and the output generates *C*_2_/2 single-channel feature maps. The expressions for each are as follows:

(5) $$Y_{SC}=X*K_{SC},Y_{SC}\in R^{\frac{1}{2}C_{2}\times H\times W},$$

(6) $$Y_{DSC}=Y_{SC}*K_{PW},Y_{DSC}\in R^{\frac{1}{2}C_{2}\times H\times W}$$

where $Y_{SC}$ is the output of SC, $Y_{DSC}$ is the output of DSC, and * represents the convolution operation.

The input feature map *X* in GSConv is divided into two parts: the main branch uses SC to extract channel-dense features, and the auxiliary branch uses a large kernel DSC to capture spatial information. The outputs of the two branches are spliced in the channel, and a shuffle uniformly fuses the channel messages to obtain a feature representation that is closer to SC but computed for only 50% of it. Finally, the output of GSConv is calculated as follows:

(7) $$Y_{GSConv}=Shuffle\left(Y_{SC},Y_{DSC}\right),\;Y_{DSC}\in R^{C_{2}\times H\times W}$$

The VoV-GSCSP module utilizes GSConv to build a complex module, as shown in Figure 4, which divides the input feature map into two parts, with the primary path extracting deep features via GS Bottleneck and the secondary path directly retaining part of the input as cross-stage information. This structure reduces redundant computation and enhances feature reuse. VoV-GSCSP is computationally efficient, about 20% faster than traditional CSP, and has enhanced feature expression capability, improving detection accuracy.

### 3.4. Dynamic Detection Head

**Figure 8:**
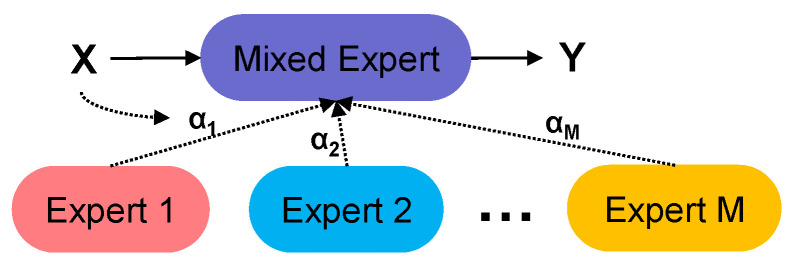
The structure of the dynamic detection head core modules.

The dynamic convolution (Dy_detect) [27] module is based on the principle of dynamic coefficient generation, which allows the model to select parameters to keep the calculation low. The convolutional layer, MoE, of the dynamic detection head is shown in Figure 8, where is *M* independent convolutional kernels. Dynamic weights α*_i_* are generated by first compressing the input features *X* by global average pooling and then generating expert weights $\alpha^{\prime }$ by a two-layer MLP and then converting $\alpha^{\prime }$ to a probability distribution by Softmax normalization and at the same time guaranteeing that $\sum_{i=1}^{M}\alpha_{i}=1$ to achieve adaptive allocation of the expert weights as follows:

(8) $$z=\frac{1}{H\times W}\sum_{i=1}^{H}\;\sum_{j=1}^{W}\;X_{c,i,j},z\in R^{C_{in}},$$

(9) $$\alpha^{\prime }=W_{2}\cdot ReLU(W_{1}\cdot z+b_{1})+b_{2},\alpha^{\prime }\in R^{M}\;,W_{1}\in R^{{d\times C}_{in}},W_{2}\in R^{M\times d}$$

(10) $$\alpha_{i}=\frac{e^{\alpha_{i}^{\prime }}}{\sum_{j=1}^{M}\;e^{\alpha_{j}^{\prime }}},\alpha \in R^{M},$$

where z is the value of the global average pooling, and $\alpha^{\prime }$ demonstrates the two-layer MLP, *d* is the hidden layer dimension, and *M* are independent convolution kernels. Finally, the exclusive convolution kernel W′ for the current input based on the weights α_i_ can been calculated as follows:

(11) $$W^{\prime }=\sum_{i=1}^{M}\alpha_{i}W_{i}\;,$$

(12) Y=X∗W′ ,

When the number of experts increase, the number of parameters expands exponentially, but FLOPs increases little by generating dynamic weights that accounts for a very low proportion of the total computation. For the background region of such simple inputs, only a small number of experts are activated. For the edges or texture fusion of the tomato surface defects, multi-experts are activated. This way achieves decoupling between the parameters and the computational complexity to reduce unnecessary computation and can be efficient in completing the detection task.

## 4. Results and Discussion

### 4.1. Software and Hardware Configuration

The CPU model used for the tomato surface defect detection model training in this paper’s experiments is the Intel(R) Xeon(R) Platinum 8352V CPU @ 2.10 GHz, with 50 G of running memory; the GPU model is the NVIDIA GeForce RTX 4090, with 24 G of graphics memory; and the operating system is Ubuntu 22.04. The relevant environment configurations are Python 3.12, PyTorch 2.4.0, and CUDA 12.1. The main configuration for the proposed YOLO-RGDD during training is listed in Table 1.

### 4.2. Evaluation Metrics

There are some key metrics for evaluating the model’s performance on tomato defect detection. Precision (*p*) and recall rate (*R*) are usually used for single class labels, and they are calculated as follows:

(13) $$p=\frac{TP}{TP+FP}$$

(14) $$R=\frac{TP}{TP+FN}$$

where *TP*, *FP*, and *FN* are the number of true positives, false positives, and false negatives, respectively.

Since this tomato surface defect detection relies on a multi-classification model, the following evaluation metrics are proposed to reflect the overall performance of the model: *mP* (mean precision), *mR* (mean recall rate), and *F1*-score. They are defined as:

(15) $$\mathrm{mP}=\frac{1}{C}\sum_{i=1}^{C}\;p_{i},$$

(16) $$\mathrm{mR}=\frac{1}{C}\sum_{i=1}^{C}\;R_{i},$$

(17) $$F1=\frac{2\times mP\times mR}{mP+mR},$$

where *C* is the number of categories, and *i* is the number corresponding to the category. The *p* and *R* of each category are calculated according to the single-category as Equation (13) and Equation (14), respectively.

Another three metrics, misclassification rate (MR) [28], false positive rate (FPR), and false negative rate (FNR) [29], are used to evaluate the performance of models under the situation of misdetection and omission. The three metrics are calculated as follows:

(18) $$\mathrm{MR}=\frac{\mathrm{FP}+\mathrm{FN}}{\mathrm{TP}+\mathrm{TN}+\mathrm{FP}+\mathrm{FN}},$$

(19) $$\mathrm{FPR}=\frac{FP}{TP+FP},$$

(20) $$\mathrm{FNR}=\frac{FN}{TP+FN},$$

For the determination of the correct label, the traditional method is to be used when the intersection of the prediction frame and the real frame is greater than a certain threshold, and then the judgment of whether the label category is the same or not is carried out, and then the judgment of whether the confidence level is greater than the set threshold is calculated and is only recognized as correct if they are all satisfied. In addition, the prediction box and the real box are in one-to-one correspondence. For the shape of the detection box, Liu [30] and others reasonably proposed a new circular bounding box (C-Bbox) instead of the traditional rectangular bounding box (R-Bbox) for tomatoes.

**Figure 9:**
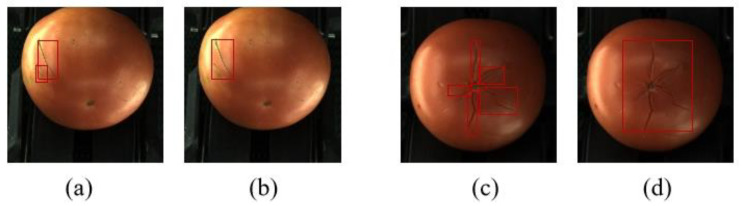
Comparison of different frames: (**a**) multi-detection frame for scars, (**b**) single-detection frame for hyperplasia, (**c**) multi-detection frame for gaps, (**d**) single-detection frame for gaps.

In the detection of tomato surface defects in this experiment, if there are two labels of the same category that are very close in distance, it will lead to a mixture of large and small boxes in the artificial real box or detection box, which will affect the authenticity of the evaluation. The specific situation is shown in Figure 9. The first group (a) and (b) is scars connected together, and the second group (c) and (d) is the connection of the gaps. In terms of the growth mechanism, scars grow irregularly and in a strip-like manner, which can lead to the situation of not being able to clearly identify whether the scars are connected or not. However, it does not affect the fact that this area is a defect of this type. In the traditional IoU standard, if (c) and (d) are true and detected frames, respectively, there are five missed detections and one wrong detection. In view of the fact that they may be combined as one label or separated as multiple labels in both manual labeling and machine detection, inspired by Liu [30], we propose a new discriminative criterion, IoM, to replace the traditional IoU in the detection frame comparison session in order to better determine whether the prediction frames correctly predict the various types of defects on the tomato.

IoU is the intersection of the area of the real frame and the area of the detected frame over their concatenation. Similarly, IoM is the smaller of the two frames at the intersection of the real frame area and the detected frame area, and it can be expressed by the formula as follows:

(21) $$IoU=\frac{A_{pred}\;\cap \;A_{gt}}{A_{pred}\;\cup \;A_{gt}},$$

(22) $$IoM=\frac{A_{pred}\;\cap \;A_{gt}}{{min(A}_{pred},\;\;A_{gt})},$$

where $A_{pred}$ denotes the predicted frame area, and $A_{gt}$ denotes the real frame area. The two schemes of IoU and IoM for the YOLO12s model were used for frame comparison, respectively, and the results of the testing set are listed in Table 2. It is clear that the adoption of the IoM compared to the IoU criterion improves the evaluation indexes *mP* compared to 2.9%, *mR* compared to 2.8%, and *F1* compared to 2.9%. On the other hand, the adoption of the IoM compared to the IoU criterion reduces the evaluation indexes MR compared to 4.4%, FPR compared to 2.9%, and FNR compared to 2.8%.

**Figure 10:**
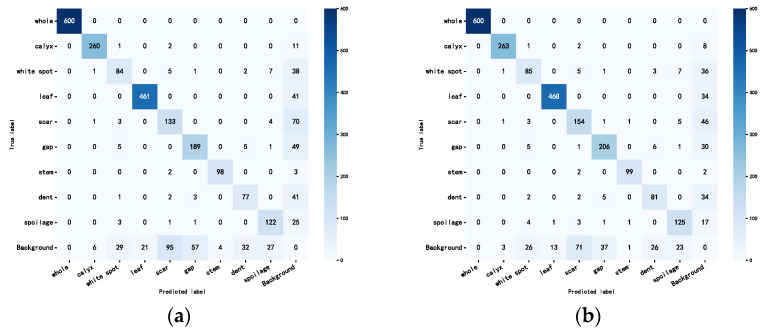
Confusion matrix plots for the test set of YOLOv12s models under IoU and IoM standards: (**a**) IoU standard, (**b**) IoM standard.

Figure 10 shows the confusion matrix diagrams of YOLO12s with IoM and IoU, respectively. It can be seen that there is a great improvement in the recall rate for the two categories of scar and gap, and the problem of misjudgment caused by the large and small boxes in the original diagrams is solved, so the evaluation indexes using the IoM criterion are more realistically reflective of the detection situation; therefore, the experiments after the study were conducted to evaluate the model comparing the real box and the predictive frames are all based on IoM as the standard.

### 4.3. Comparison of Different Detection Algorithms

To evaluate the performance of the proposed method, a group of comparison experiments was carried out under consistent experimental conditions, and the results are listed in Table 3. Fast-RCNN, SSD, and Efficientdet have worse detection performance than the YOLO series, while Efficientdet has the smallest computational complexity with 6.1 GFLOPs. Among the first three models, SSD has the worst performance with the lowest mP and mR and the highest MR and FNR. Although YOLO-NAS [31] has the smallest computational complexity among the YOLO series, its defect detection precision is the worst. YOLOv9s has the largest computational complexity of 27.6 GFLOPs, with an unsatisfactory recall of 81.0%. YOLOv12s balanced the precision and recall rate and achieved a relatively outstanding detection performance among the YOLO series. It is noteworthy that the proposed YOLO-RGDD demonstrates excellent performance, with mP, mR, and F1 reaching 88.5%, 85.7%, and 87.0%, respectively, which are 4.1%, 2.0%, and 3.5% higher than those of the standard YOLO12s, respectively. YOLO-RGDD has the smallest computational complexity of the s-series at 16.1 GFLOPs and the lowest MR of 15%, FPR of 11.5% and FNR of 14.3%.

**Figure 11:**
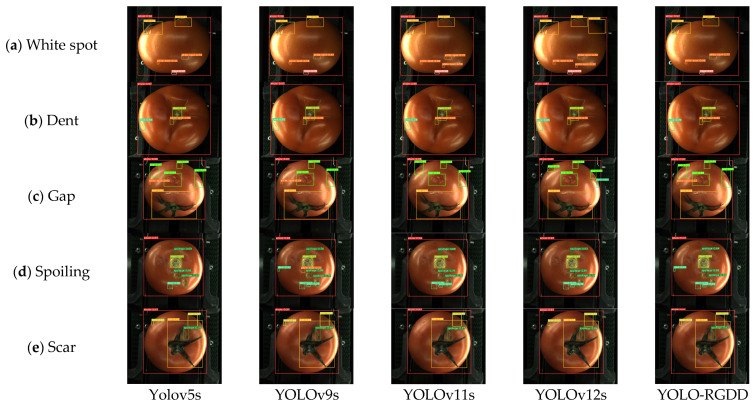
Results of different surface defect categories with different sizes by YOLO series.

Figure 11 illustrates the physical detection diagrams for the YOLO series, where the different defect categories and varying degrees of defects show significant differences between the evaluated models. For the safety labels, including whole, leaf, stem, and calyx, all models were successfully detected. For the white spot defects on the tomato surface in Figure 11a, YOLOv11s and YOLOv12s have misdetections, and the average confidence level of the proposed model YOLO-RGDD for white spot is 0.73, which is higher than that of YOLOv5s (0.54) and YOLOv9s (0.48), and in Figure 11b, the detection results of each are consistent. In Figure 11c, with more gaps, YOLOv9s has one missed detection of a gap, and YOLO-RGDD has the highest mean confidence (0.71) and correctly predicts the white spot with less distinctive features in this figure, which is missed by YOLOv11s and YOLOv12s. In Figure 11d, YOLO-RGDD maintains the highest confidence level for all spoilage defects, which are all correctly predicted, and accurately predicts the depression in the figure. At the same time, all other models have misdetections and false negatives. For the scar in Figure 11e, YOLOv11s has one missed detection. The previous models have misdetections and omissions to varying degrees. In contrast, the YOLO-RGDD model predicts the highest precision and recall rate, which is greater than most models. However, not every defect label confidence remains the highest, indicating the high reliability of the model.

### 4.4. Ablation Experiment

To verify the finiteness of the module proposed in this paper in different categories and degrees of surface defects and to exclude the mutual interference between modules, the evaluation indexes based on YOLO12s and between each module were set up. Since dysample is also in the Neck network, it was formed into a DSN module together with VoV-GSCSP and GSConv. In Table 4, the evaluation indexes are mP, mR, F1, GFLOPs and parameters, where GFLOPs and parameters represent the model computation and parametric quantities, respectively.

The ablation experiments show that the computational complexity of the original YOLOv12s model is 21.4 GFLOPs with 9.3 M parameters, and the mP, mR, and F1-scores are 83.4%, 83.7%, and 83.5%, respectively. Although it maintains a strong performance in terms of precision and recall rate, the computational complexity and the model parameters are larger, and there is a large room for improvement in the F1-score. After adding the DSN module, mP and mR are significantly improved by 2.7% and 1.3%, respectively, and 0.4 GFLOPs reduce the computational complexity, but the model parameters increase by 0.1 MB, which is because GSConv combines the SC and DSC to increase the number of channels so that the total number of parameters rises. The reduction of computational complexity is due to the efficient computation of channel-by-channel convolution by the DSC. The RFEM module enhanced feature extraction using its multi-branch expansion convolution design: The F1-score increased by 0.6% compared to YOLOv12s while reducing the computational complexity; and the computational complexity and the parameters are reduced by 2.4 GFLOPs and 1.2 M, respectively. Dy_detect reduces the unnecessary computation expenditure by introducing multiple expert numbers and dynamic detection, and the F1-score is increased by 0.4% while reducing the computational complexity (2.0 GFLOPs) and the model parameters (0.4 M).

Combining the modules to observe their synergistic benefits to the model and combining the DSN and RFEM modules, the model’s mP, mR, and F1-scores reach 86.7%, 86.9%, and 86.7%, respectively, which are at least 0.6%, 1.9%, and 1.1% higher than the original model and the three modules added alone but are accompanied by an increase in computational complexity and model parameters, which increase by 2.0 GFLOPs and 0.3 M, respectively. Combining the two modules, DSN and Dy_detect, the mP, mR, and F1 indexes are higher than the original model or the single-module model, and the computational complexity reaches a relative minimum of 18.8 GFLOPs. Dy_detect can balance the number of model parameters and the detection effect for processing the parameter increase of its dynamic detection header. The combined F1-score of the RFEM and Dy_detect detection methods is 84.8%, which is not as good as the other two dual-module combinations, but the computational complexity and the model parameters are greatly reduced by 4.7 GFLOPs and 1.3 M relative to the original model, respectively. This proves that DSN works well for the model, although it increases the number of parameters.

When fusing DSN, RFEM, and Dy_detect modules, the model achieves a high precision detection performance, with mP, mR, and F1-scores of 88.5%, 85.7%, and 87.0%, respectively. The computational complexity is smaller than that of the previous minimum computation model by 0.6 GFLOPs. It is even lower than that of the original model by 24.8%. Compared with the model without the addition of the Dy_detect module, the computational complexity and the model parameters are 31.2% and 17.7% lower than the original model, which better proves that Dy_detect plays a major role in balancing the increased number of parameters while upgrading the model. The experiments show that the improved scheme with the multi-branch dilated convolutional design, Slim-Neck design, and dynamic detection head effectively and comprehensively improves the precision and recall of the model while significantly reducing the model computational complexity, providing a reliable path for deploying tomatoes to the end of the conveyor belt and thus screening surface defects.

**Figure 12:**
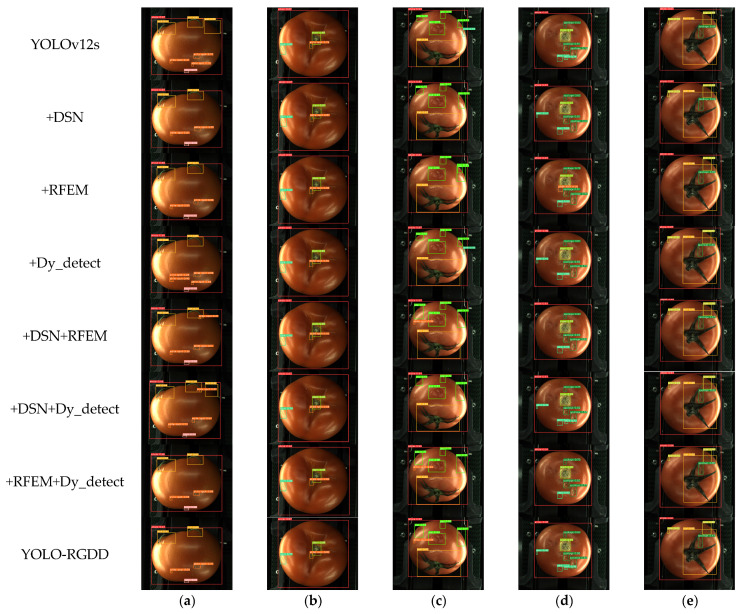
Detection results of YOLOv12s with different modules: (**a**) white spot-defective tomato, (**b**) dent-defective tomato, (**c**) gap-defective tomato, (**d**) spoiling-defective tomato, (**e**) scar-defective tomato.

Figure 12 shows the multiple defect detection of tomatoes with the addition of different modules based on YOLO12s. In Figure 12a, most of the models successfully detect white-spot defects, and YOLO-RGDD achieves the largest average confidence (0.73) for white spots; YOLO12s and YOLOv12s+DNS show varying degrees of misdetection for detecting white spots in the background. In Figure 12b, all the models detect correctly, with an average confidence of 0.8 and above for dents and white spots on the surface of tomatoes. For the gap defect in Figure 12c, DSN+RFEM, DSN+Dy_detect, RFEM+Dy_detect, and YOLO-RGDD all predicted correctly, and DSN+RFEM achieves the highest confidence of 0.77, and the remaining four models misidentify the gap as a dent or miss the gap. Additionally, they miss a white spot in Figure 12c along with DSN+ Dy_detect. In Figure 12d, DSN+Dy_detect and YOLO-RGDD all detect correctly, although the rest of the models mainly have different degrees of missed detection of the dent defects. DSN and Dy_detect detect the insignificant dent defects on the left side in the single module. In Figure 12e, all models detect correctly except for RFEM’s module, which misses a scar; and DSN+Dy_detect has the highest confidence level of 0.74 for the detected scar defects. In summary, the multi-module combination model is better overall than the single-module model, and the overall precision of YOLO-RGDD remains, which can also reflect the advantages of YOLO-RGDD over the other modules to be integrated.

### 4.5. Discussion

To improve the accuracy of defect detection and the efficiency of model operation, this study proposes an improved YOLO-RGDD for five surface defects of tomatoes. In comparison with the initial YOLOv12s, the proposed YOLO-RGDD achieves an overall improvement in precision, recall rate, and F1-score by 4.1%, 2.0%, and 3.5%, respectively, and the misidentification rate (MR), false positive rate (FPR) and false negative rate (FNR) decreased by 3.5%, 5.1% and 2.0%, respectively. On the other hand, GPLOPs and parameters were reduced by 5.3 and 1.4 M, respectively. These results indicate that the proposed model improved the detection performance while decreasing complexity.

Compared to Shi et al.’s research [32], this study provided a clear identification of the defect categories and detection accuracy rather than just theoretical method introductions. The proposed detection model proposed in this paper can effectively identify five types of defects on the surface of tomatoes with a low model complexity. The RFEM module was introduced to enhance the feature extraction for small targets while reducing the computational complexity and parameter counts, and the Dysample and Slim-Neck structure in the neck part were introduced to enhance the detection of subtle defects, better process the feature information, and improve the detection precision and recall rate, and the Dy_detect solves the problem of parameter quantity enhancement brought by the Slim-Neck framework, which greatly reduces the computational complexity and improves the performance of the model at the same time.

Due to the constant conveyor belt environment with a single detection background, the dynamic convolution of Dy_detect greatly reduces the computation of the background, i.e., through the dynamic weighting, it reduces the computational complexity on the background features and improves the detection efficiency, which is suitable for deployment in agricultural production, especially in automated equipment indoors with such a single environmental background.

## 5. Conclusions

In this study, a novel model, YOLO-RGDD, for detecting the surface defects of tomatoes is proposed by introducing RFEM, Dysample, Slim-Neck, and Dy_detect to YOLOv12s. The IoM criterion is designed to label detection results accurately. The proposed model enhances the detection of tiny defects, with an average precision of 88.5%, which is higher than other series of YOLO models and YOLOv12s with other modules added. The general increase in the confidence level, to some extent, can also indicate the enhancement effect of the proposed model on overall defect detection. In addition, the proposed model is the smallest among the s-series in terms of computational complexity, which is 5.3 GFLOPs lower than the base YOLOv12s, contributing to the lightweighting of the model.

However, there are some limitations of the proposed model for detecting defects, such as requirements for the stability of light sources in the collection environment, which constrain the application of this model to other outdoor multi-target detection scenarios. In the future, we will undertake studies under different lighting environments to improve the robustness of the model.

## Figures and Tables

**Table 1:** Training configuration for the proposed YOLO-RGDD.

Image Size	Batch Size	Epochs	Optimizer	Learning Rate
640	32	600	SGD	0.01

**Table 2:** Performance metrics for the test set of YOLOv12s models under IoU and IoM standards.

	mP (%)	mR (%)	F1 (%)	MR (%)	FPR (%)	FNR (%)
IoU	80.5	80.9	80.6	22.9	19.5	19.1
IoM	83.4	83.7	83.5	18.5	16.6	16.3

**Table 3:** Performance of different models for detecting surface defects of tomatoes.

Methods	mP (%)	mR (%)	F1 (%)	MR (%)	FPR (%)	FNR (%)	GFLOPs
Fast-RCNN	79.0	75.4	77.0	25.0	21.0	24.6	121.4
SSD	79.2	71.2	74.6	26.7	20.8	28.8	75.6
Efficientdet	80.4	77.3	78.7	23.1	19.6	22.7	6.1
YOLOv5s	85.7	80.6	82.9	19.6	14.3	19.4	24.2
YOLO-NAS	79.2	82.6	80.8	22.8	20.8	17.4	12.5
YOLOv9s	84.4	81.0	82.5	19.3	15.6	19.0	27.6
YOLOv11s	85.0	81.1	82.8	18.9	15.0	18.9	21.7
YOLOv12s	83.4	83.7	83.5	18.5	16.6	16.3	21.4
YOLO-RGDD	88.5	85.7	87.0	15.0	11.5	14.3	16.1

**Table 4:** Performance metrics of tomato surface defect detection based on the YOLOv12s model by increasing or decreasing modules through control variables.

YOLOv12s	DSN	RFEM	Dy_Detect	mP (%)	mR (%)	F1 (%)	GFLOPs	Parameters (M)
√				83.4	83.7	83.5	21.4	9.3
√	√			86.1	85.0	85.5	21.0	9.4
√		√		85.2	83.2	84.1	19.0	8.1
√			√	84.6	83.5	83.9	19.4	8.9
√	√	√		86.7	86.9	86.7	23.4	9.6
√	√		√	87.3	86.5	86.8	18.8	9.1
√		√	√	86.2	84.3	84.8	16.7	8.0
√	√	√	√	88.5	85.7	87.0	16.1	7.9

## Data Availability

The original contributions presented in the study are included in the article. Further inquiries can be directed to the corresponding author.
